# DHEA and frontal fibrosing alopecia: molecular and physiopathological mechanisms*

**DOI:** 10.1590/abd1806-4841.20165029

**Published:** 2016

**Authors:** Neide Kalil Gaspar

**Affiliations:** 1 Universidade Federal Fluminense (UFF) - Niterói (RJ), Brazil

**Keywords:** DHEA, FFA, Frontal fibrosing alopecia, Alopecia, Dehydroepiandrosterone, PPAR gamma, Transforming growth factor alpha

## Abstract

The transforming growth factor-beta 1 (TGFβ1) promotes fibrosis, differentiating epithelial cells and quiescent fibroblasts into myofibroblasts and increasing expression of extracellular matrix. Recent investigations have shown that PPAR (peroxisome proliferator-activated receptor*) is a negative regulator of fibrotic events induced by TGFβ1. Dehydroepiandrosterone (DHEA) is an immunomodulatory hormone essential for PPAR functions, and is reduced in some processes characterized by fibrosis. Although scarring alopecia characteristically develops in the female biological period in which occurs decreased production of DHEA, there are no data in the literature relating its reduction to fibrogenic process of this condition. This article aims to review the fibrogenic activity of TGFβ1, its control by PPAR and its relation with DHEA in the frontal fibrosing alopecia.

## INTRODUCTION

The frontal fibrosing alopecia (FFA) is a form of alopecia manifested in women, very rarely in men, and it begins in frontal hairline, progressing in the lateral and posterior direction, with permanent loss of hair follicles and may reach also the eyebrows and other areas.^1^

Of unknown etiology and treatment with unsatisfactory results, it was recently compared to a similar type of hair loss found in mutant animals presenting changes in functions of specific intranuclear receptors.

The occurrence of this event almost exclusively in women, and those in the pre or post-adrenopause period, raises the need to relate the hormonal changes concerning the development of alopecia.

Even maintaining the present article under "review article" designation, only the essential facts to the current conceptualization of the disease are presented, leaving more space for the aspects that are considered relevant:

- Is the reduction of the local activity of dehydroepiandrosterone (DHEA) responsible for fibrosis and loss of hair follicles?
- May DHEA for topical use be useful in its treatment?

## HISTORY

In 1994, Steven Kossard described a new and peculiar hair loss called the frontal fibrosing alopecia of women (FFA).^1^ Its histology was similar to lichen planopilaris (LPP), without the occurrence of signs of lichen planus in the rest of the tegument. Sixteen years before Josefowicz identified a mutant species of mouse, named Asebia, with alopecia and atrophy of sebaceous glands.^2^ Genetic aspects related to alopecia in these mice were mapped in a second mutant animal, the Asebia 2, spontaneously developed in laboratory and with extreme atrophy of sebaceous glands and hair follicles progressing to fibrosis.^3^

The genotype of these animals, with changes resulting from chromosome 11 mutations, was identified, evidencing the entire follicular degeneration process.^4^ The fact that the activity of PPAR α and γ was essential for the differentiation and functions of sebocytes was followed by identification of malfunctions of these receptors in Asebia 2.^5^

It has been shown that deletion of PPARγ in the follicular bulge generated changes similar to LPP.^6^ Similarly, the finding that the functional maintenance of hair follicles depends on the presence of multipotent cells in the bulge demonstrated the importance of investigating the effect of stimulant drugs of PPARs in the treatment of alopecia.^7^

The fibrosing inflammatory follicular changes in Asebias were reviewed and appraised as not autoimmune. Stem cell markers aberrations in the follicles during the cell cycle were demonstrated and accountable for the changes presented.^8^ The exact etiopathogenic relation between FFA and LPP is not established, and the various tested treatments have only stopped the process of expanding in inconstant percentage of cases. ^9^

## EPIDEMIOLOGICAL AND CLINICAL ASPECTS

Re-evaluating 355 cases in a multicenter study, only 12 were men, 49 were women with early menopause and 49 were premenopausal women. In 37% patients, alopecia was found to be severe. Mean age was 61 years (between 23 and 86 years).^10^

FFA is characterized in its early stages by retraction of the previous limit of hairline, with evolution in lateral and posterior direction, and may present erythema, micropapules and hyperkeratosis in the follicles. Mild skin atrophy, itching and burning sensation may occur in some patients, as well as partial and even total loss of eyebrows. The final result is a scarring aspect in the affected area. The progression of the process is slow and spontaneous remission may occur. Lesions in other areas of the scalp have been described. Loss of eyebrows occurred in 73% of patients, of eyelashes in 3%, and of hair body in 25% of 60 patients.^11^ In a series of 16 cases, 8 presented loss in eyebrows and 6 presented axillary alopecia.^12^

## DERMATOSCOPIC DIAGNOSIS

Dermoscopy can indicate the area of biopsy and should be performed where there is perifollicular concentric scales that are less scaly than LPP. Perifollicular erythema is indicative of disease progression and may be present in other scarring alopecia, such as discoid lupus erythematous and LPP. Keratosis pilaris is also common to discoid lupus erythematosus.^13-15^ In the lesions of 79 women, 72.1% showed hyperkeratosis, 66.3%, perifollicular erythema, and 44.8%, follicular plugs.^16^ Erythema and perifollicular scales indicate progressive disease activity.^14^ There is progressive loss of follicles with perifollicular erythema with and without scales. The absence of follicular ostia is the ultimate characteristic feature.^17^ Within a 249 case review, there was presence of follicular hyperkeratosis in 89% of them, perifollicular erythema in 77%, isolated hair in 67.9%, diversity of diameters in 45%, scarring white patches in 22.3% and yellow dots in 21.9%.^18^

## HISTOPATHOLOGICAL DIAGNOSIS

Biopsy reveals the presence of lymphocytic infiltrate involving the bulge and the infundibulum. Apoptotic cells in the outer sheath of the rod and concentric fibrosis around the follicle with decrease in the follicle number, replaced by fibrous tissue, are present.^19^ The location of the infiltrate is centered in the bulge region.^20^ These aspects occur in LPP and although there is no clear tissue difference between LPP and FFA, there is milder inflammation and apoptosis in FFA than in LPP.^20,21^

## TREATMENT

Stabilization of the anterior limit of the implant can be achieved with intralesional corticosteroids in most patients.^22^ Stand-alone topical corticosteroids are not effective.^23^ Evaluating 15 patients, hydroxychloroquine at a dose of 400 mg/day led to a reduction of signs and symptoms in 11 of them, reached between 6 and 12 months of administration.^24^

Among the 22 cases of LPP with pioglitazone ministration, only four are presented as FFA, and three of them reacted to treatment.^25^

Systemic anti-inflammatory drugs, tacrolimus, tetracyclines, cyclosporine, mycophenolate mofetil, combined or not with other substances, have been used in small case series, with varied responses.

Antimalarials, intralesional steroids and antiandrogens are the most often cited as being able to stop the progression of FFA.^10,26-28^

In the extensive review of Vano-Galvan *et al.*, the antiandrogens have the best results in controlling the progression of the disease. In the experience of these authors, intralesional corticosteroids associated with antiandrogens were useful in the presence of erythema or follicular hyperkeratosis.^10^ Only this process of stabilization is reported in response to different treatments tested.

The transplantation of hair follicles can have results in cases of disease in remission. ^29^

## PHYSIOPATHOLOGY: MOLECULAR MECHANISMS

Integrity and functions of sebaceous gland are essential for the development and activity of hair follicles^4^ stimulated by PPARγ.^5^ Mice depleted of the gene of this receptor lose the stem cells of the bulges and develop fibrosing alopecia.^5,6^ PPARγ is indispensable for the maintenance of stem cells of functional epithelium in hair follicles.^5^ Deletion of the PPARγ gene in the bulge area of the hair resulted in a skin process is similar to LPP alopecia.^6^ The location of the inflammatory infiltrate is typically centered on the bulge region.^21^

Patients with LPP have changes of expression of this gene, indicative of defect in lipid metabolism and of peroxisomes biogenesis.^7^ The comparative analysis of biopsies in areas with scarring alopecia and unaffected areas of patients revealed a decrease in expression of genes required for lipid metabolism and biogenesis of PPAR. In lesions of FFA, there is increasing loss of these receptors, pro-inflammatory lipid accumulation, inflammatory cell infiltration and destruction of follicular units.^7^

There is considerable evidence on the role of PPARγ and its ligands in inhibiting fibrosis in different organs and tissues.^30-33^ These nuclear receptors act as lipid sensors to modulate gene expression. Thus, they participate of the main metabolic and inflammatory tissue regulations, with extensive physiological and pathological consequences, as well as in important regulatory processes of cell fate.^34^

Three receptor subtypes, α, β and γ, phylogenetically related, but encoded by different genes, have been identified with predominant roles in the PPAR family. PPARγ is more expressed in the sebaceous glands and adipose tissue, and less frequently in the colon, adrenal glands, spleen, and skin. In these sites, PPARγ mediates storage of fatty acids. It also regulates lipogenesis, local inflammatory and carcinogenic response, sebocytes and keratinocytes differentiation, wound healing and positive response to the ultraviolet irradiation.^35-39^

PPARs are abundant in the adult epidermis and their activation induces intense proliferation of peroxisomes. A growing body of research indicates that the γ subtype of the PPAR (PPARγ) is the key to control the activity of TGFβ1 in suppressing fibrogenesis. The imbalance of its control in skin fibroproliferation is responsible for excessively fibrous activities.^38^

TGFβ1 is a pro-oncogenic cytokine that leads to epithelial/mesenchymal transdifferentiation and to fibroblast/myofibroblast transition, a crucial event for collagen synthesis.^39-42^ The development of fibrosis after tissue injury requires activation of TGFβ1.^42^

Currently, it is accepted that the epithelial/mesenchymal transition, primarily described in critical stages of embryogenesis, may occur both in development of tumors and in non-tumor tissues, by the action of TGFβ1, together with different local growth factors. ^41^ This transition, concomitant with increased TGFβ1, is carried out via SMAD protein, transducer of the extracellular signal, increasing the production of reactive oxygen species (ROS) in the cytoplasm and mitochondria.^42,43^

These facts have led researchers to experiment with different drugs with the potential to stimulate PPARγ in vitro in the treatment of LPP and also aiming to develop therapeutics of fibrotic processes in different organs.^28,30-33,35-37,43-46^ However, PPAR activity in hair follicles have not been investigated in relation to hormonal changes typical of adrenopause.

In recent years, various investigations have established that stimulation of endocrine PPARs is especially related to the activity of dehydroepiandrosterone. DHEA and its sulfated product (DHEAS) are the most abundant circulating steroid hormones in humans. Its production in women have higher levels between 25 and 30 years, decreasing after reaching this stage and reaching adrenopause at 60 years, with only 10% to 20% of peak levels.^47^

DHEA performs immunomodulatory role and is essential for the nuclear receptor PPAR in the transcription of genes, in fat metabolism, and in mitochondrial activity.^48-49^ The imbalance of its functions can trigger inflammatory and autoimmune changes.^48^

DHEA is synthesized in several somatic sites, such as adrenal cortex, ovarian theca and brain. Skin cells have all the biochemical structure necessary for the production of glucocorticoid, estrogen and androgens. This synthesis can be performed from systemic origin precursors or, alternatively, through conversion of cholesterol to pregnenolone and its subsequent transformation to biologically active steroids.^50^ Disorders in cutaneous steroidogenesis may have local and systemic effects, triggering inflammatory or autoimmune processes.^50^

Cutaneous steroidogenesis, which mainly occurs in the sebaceous gland, has a distinct pattern of evolutionary topographic behavior during the aging process, as evidenced by studies conducted only in males. DHEA and androstenedione concentrations measured in the liquid content of suction blisters in different areas of the body were compared to the blood level in young men (27,8 years) and elderly subjects (62.6 years). Increased concentration of DHEA was found, and not of androstenedione, in the liquid of the blisters of the elderly subjects, inversely to reduced blood levels. In the studied skin sites, the differences between young and old individuals were higher.^51^ Similar tissue studies in women have not been found in the literature.

The importance of the effect of DHEA on mitochondrial oxidative metabolism was stabilished by administration of this hormone in rats. These animals showed increased liver and brain metabolism with elevated brain weight without concurrent increase in body weight.^52^ The results observed in the topographic response is demonstrative of the variation in distribution of DHEA receptors in the body of these animals.

Tests with radioactive DHEA administered to dogs confirmed their higher metabolism in fibroblasts derived from dermal follicles and, among these, in dermal fibroblasts derived from the follicles of the skulls of the animals, accentuating the topographic differences of their receptors.^53^

The relation between the activity of DHEA and FFA is also suggested by the reduction of DHEA levels in different conditions characterized by fibrosis, such as occurs in idiopathic pulmonary fibrosis.^47^ A comparison of the serum levels of DHEA/DHEAS of 137 patients with idiopathic pulmonary fibrosis and 58 controls showed a significant reduction of these hormones in patients, both in plasma and in the bronchoalveolar lavage fluid.

The importance of DHEA in the fibrogenic process was also demonstrated by the addition of DHEA to cultured fibroblasts obtained from bronchoalveolar lavage from patients, which resulted in the lowest differentiation in myofibroblasts and decrease in collagen production and duplication of apoptosis levels compared to cultivated ones without the addition of hormone.^47^ Idiopathic fibrosis is a disease related to aging and is characterized by expansion of myofibroblasts and lung remodeling.^47^

Epithelial/mesenchymal transition also develops in chronic asthma with great impact on the bronchial functions. Bronchial epithelial cells of the human lineage 16HBE-14, induced to fibrosis by TGFβ1, were prevented from developing it by adding DHEA to their cultivation.^38,40^

The importance of DHEA was further corroborated by the addition of supraphysiological doses of this hormone to the cultivation of HepG2 cell line of human hepatoma, with substantial response of PPAR gene expression in both the transcriptional as in post-transcriptional level.^54^

Having found that the essential fact in FFA is the dysfunction of PPARγ and that the function of this receptor is linked to stimulation of DHEA, it is vital to define its participation in the development of fibrosis in this alopecia. However, in the medical literature, there is no information on the relation between the fibrogenic process of FFA and endocrine changes of adrenopause regarding DHEA.

## DISCUSSION

The exposed aspects that relate to fibrogenic activity to reduction of PPARs function and their dependence of the activation by DHEA, lead us to suppose that the reduced activity of this hormone, characteristic of the phase in which this alopecia occurs, correlates directly to fibrogenic inflammatory process of FFA.

Knowing that the activity of a hormone depends on both its blood content as the functional integrity of specific cellular receptors, and that these are presented in topographic and individual variation, the simple blood hormonal evaluation does not reflect the degree of their local operations. Thus, verification of their functions in the lesional areas becomes necessary.

It is possible that the benefits obtained by the administration of 5α-reductase inhibitors in the FFA are the result of DHEA impediment in reaching its final conversion to dihydrotestosterone.

The exact relation between FFA and LPP is not established. Although Samrao^23^ has reported the presence of lichen planopilaris in 14% of 36 cases, MacDonald *et al.* found it only in 1.6% in a series of 60 cases of FFA.^11,23^ Although in some patients with typical clinical aspect of FFA occur the presence of lichen planopilaris, it is essential to answer the following questions:

- Why almost all cases of FFA are presented in adrenopause women?
- What are the differences between the pathogenic processes underlying to LPP and FFA?
- If in FFA the hypothesis of this article is confirmed, should it also be extended to LPP lesions?

## CONCLUSIONS

1. PPARγ is the main regulator of lipid cell metabolism and sebocytes development. The reduction of its activity is responsible for fibrosis;
2. DHEA is an important stimulator of PPARs;
3. Reduction of DHEA is observable in some diseases characterized by fibrosis;
4. Frontal fibrogenic alopecia typically develops in the woman life period in which production of DHEA is decreased;
5. It is possible that the reduction of the local activity of DHEA is responsible for follicular fibrogenic process of this alopecia;
6. It is also possible that the better understanding on the variation of topography dynamic of tissue activity of DHEA changes therapeutic strategies in other conditions characterized by fibrosis.
